# Accurate device-independent colorimetric measurements using smartphones

**DOI:** 10.1371/journal.pone.0230561

**Published:** 2020-03-26

**Authors:** Miranda Nixon, Felix Outlaw, Terence S. Leung

**Affiliations:** Department of Medical Physics and Biomedical Engineering, University College London, London, United Kingdom; Nanjing University of Information Science and Technology, CHINA

## Abstract

Smartphones provide an ideal platform for colorimetric measurements due to their low cost, portability and image quality. As with any imaging-based colorimetry system, ambient light and device variations introduce error which must be dealt with. We propose a novel processing method consisting of a one-time calibration stage to account for inter-phone variations, and an innovative use of ambient light subtraction with image pairs to account for variation in ambient light. Data collection is kept very simple, making it particularly useful for use in the field, since nothing additional is required in the images. Ambient subtraction is first demonstrated for a range of colors and phones (Samsung S8 and LG Nexus 5X), and the Subtracted Signal to Noise Ratio (SSNR) is defined as a metric for assessing whether an image pair is appropriate at the time of image capture. The experimentally determined SSNR threshold below which to suggest retaking the images is 3.4. The classification accuracy for results using the proposed calibration pipeline is then compared to the simplest image metadata-based alternative and is found to be greatly superior. Finally, a custom colorcard is shown to improve the accuracy of device-independent results for known smaller ranges of colors over a standard colorcard, making this a possible application-specific modification to the overall processing pipeline.

## Introduction

The desire for quantitative measurements of color exists across many fields. For example, quantifying colorimetric urine tests for measurements of pH and glucose [1–4] or determining saliva alchohol concentration [5]. As well as test strips, within medicine there are further applications which aim to quantify colorimetric biomarkers of the human body to detect conditions such as jaundice [6–8], anaemia [9] and the eye condition anterior blepharitis [10]. Applications continue beyond medicine, for example in testing water quality [11] or improving the rigour of marine monitoring [12].

Commercial devices for contact measurements of color exist which are becoming more affordable, for example the X-Rite CAPSURE [13], but for many of the above applications a contact measurement is not possible. Imaging presents an attractive non-contact alternative. Digital cameras provide the best image quality, but can still be expensive and bulky to transport. Smartphones, on the other hand, are incredibly portable. Until recently, the crucial access to raw images from smartphone cameras was highly limited, but this is becoming more common. Additionally smartphones are becoming even cheaper and more ubiquitous, with over 8 billion subscriptions by 2018 [14]. With a smartphone, unlike with a digital camera, it is possible to create an app which combines the image capture with the required additional processing to produce results in real time. All of these factors, combined with the continued increase in smartphone image quality, makes them the ideal candidate for use in colorimetry.

Unlike the human visual system, cameras don’t account for changes in ambient light automatically. This means that different color values will be recorded for the same object under different lighting conditions. For consistent colorimetry, this effect must be mitigated. Previous work has utilised a variety of approaches to tackle this problem. A simple approach is to calibrate the phone before every new measurement [1], however this increases the capture time for every single capture and so is not very efficient. An alternative is to include a standardised white card in every image, using white balance to account for lighting changes [15]. This again complicates the image capture process. Another approach is to remove the ambient light entirely, and use either the flash of the phone or an alternative light source as a fixed illumination [5, 7, 11]. This does remove the impact of ambient light, but requires a custom setup for every phone, and for some applications it may not be a viable approach. It is challenging to find an approach to deal with ambient light which is both general and simple enough.

The second challenge of using imaging for colorimetric measurements is that different phones will record significantly different values even under identical conditions. The simplest approach would be to limit the technique to a single phone. However, for a method to be more broadly applicable it is better for it to work on multiple phone types. One way to tackle this problem is to use machine learning [3, 16]. This approach can deal with changes in lighting and the use of different phones in one stage, however it requires very large training sets which continue to increase in size the more general you would like to be. Additionally, depending on the particular method, it is common to require internet access to store and apply the resulting model. This is a large problem for use in low and middle-income countries, where the need for cheap and portable approaches is most high. The color science approach to deal with variations between phones is to move from values which are specific to a phone to device-independent values using a mapping. Open source software dcraw [17] uses information stored in the image metadata to do this. Metadata methods are optimised for a particular light and additionally do not account for variations between devices of the same model, which can lead to unreliable results [18]. It is also possible to account for inter-device variations by including a card with a standardised range of colors on it in every image [19] and so develop a mapping. This greatly increases the complexity of image capture, particularly for human subjects.

Here, we suggest an overall approach tackling both problems. Variations in ambient light at the image capture stage are accounted for using the technique of ambient subtraction—a pair of flash no-flash images are captured, and a subtraction removes the effect of the ambient light [20, 21]. Variations between devices are tackled by carrying out a one-time calibration for each device, resulting in a mapping which is specific to that phone and its flash illumination, and which can therefore be used on data which has been ambient subtracted. This combined approach means that data captured is consistent over different lighting environments and phones, and hence the link from measured color to the application specific scale, for example jaundice or pH, only needs to be determined once. In this paper, we describe the theory behind the two major stages and present data demonstrating the quality of the approach.

## Theory

The theory required to understand how an image is formed and recorded by a smartphone is described, highlighting the challenges which need to be overcome before reliable colorimetric measurements are possible. The two steps of our proposed pipeline which deal with these issues are then covered in more detail.

### Image formation

There are three key factors involved in forming an image, and the interplay between them determines the final image. The first factor is the illumination, or lighting, of the surroundings. The spectral power distribution of the illumination determines its color, for example the yellowness of daylight compared to the blueness of fluorescent lighting. Color constancy is an automatic property of the human visual system, whereby changes in ambient illumination color are accounted for, and our perception of colors remains stable. This is not the case for a camera, and so recorded values for an image are greatly influenced by the illumination.

The next factor in image formation is the scene that is being imaged, the actual objects of interest—this is the component of the three that we are interested in extracting information about. Elements of the scene can reflect or absorb the light. Materials that selectively absorb wavelengths modulate the light spectrum and hence appear a different color. The spectrum of light resulting from a scene will therefore depend on both space and wavelength. As well as the object’s spectral properties, the light recorded also depends on the object geometry with respect to the light source.

The third key factor in forming an image is the camera itself. The incident energy must be recorded in a reproducible way. Cameras achieve this by describing the light as a sum of three channels—red, green and blue (R, G, B from here on), similar to the human visual system. Each of the RGB channels will span a certain range of wavelengths, with a peak sensitivity in the red, green and blue regions of the visible light spectrum respectively. Combining these three factors of image formation in equation form, the values measured by a camera in each color band, *c* ∈ {*R*, *G*, *B*}, are given by

$$\begin{array}{c} f^{c}\left(x\right)=m\left(x\right)\int_{\omega }s\left(\lambda ,x\right)e\left(\lambda \right)\rho^{c}\left(\lambda \right)d\lambda \end{array}$$ (1)

where *m*(***x***) gives the geometric dependence of the reflectance, *ω* represents the visible spectrum, *s*(λ, ***x***) relates to the spectral reflectance properties of the surface, *e*(λ) is the illumination of the scene and *ρ*^*c*^(λ) is the spectral sensitivity of the camera in each color band [22].

Measuring the full RGB values for every sensor site would be very complex and would make cameras prohibitively expensive. Instead, imaging sensors are used in combination with a pattern of red, green and blue filters which allow light of just that wavelength band through at each pixel. Each sensor then has a sensitivity determined by the filter, which can be understood as the probability for that sensor to detect a photon of a given wavelength. The filters are arranged in a pattern known as a Bayer pattern [23], and this results in a given pixel containing information about one of red, green or blue light. The spectral sensitivity of smartphones will vary between manufacturers and even between devices of the same make and model, meaning that the values recorded by two phones of identical objects under the same illumination will be different. This variation is clearly an issue which needs to be tackled for a colorimetric method to be generalisable.

From the recorded Bayer pattern image, various stages of processing are carried out automatically by the camera to yield the JPEG images we are used to seeing. These stages include interpolation to obtain an RGB value for every pixel, but also scaling of the color channels and compression. The resulting images are unsuitable for scientific use for two reasons. Firstly, the compression means that there has been a loss of information. Secondly, given that we are aiming to quantify colors, having unknown modification happening to the color channels introduces unnecessary uncertainty. Our approach is therefore based on analysis of the raw recorded Bayer pattern images.

It is clear from inspection of Eq 1 that it is not simple to separate out the influence of the lighting or the camera from the object on the final pixel values. We have therefore devised a two step process to account for their influence.

### Ambient subtraction

The overall aim of ambient light correction is to remove the effect of ambient light on the image and allow comparison of images from capture sessions under different lighting conditions. To maintain a simple procedure for image capture, we required that the method did not involve introducing anything additional into each image. There are many approaches which attempt ambient correction by first estimating the illumination and then removing its effect [24–26]. These approaches assume access to only a single image of the scene, however using a smartphone it is simple to capture a pair of images which expands the techniques available. We have previously introduced a novel approach using subtraction of flash no-flash pairs of images [21]. A pair of images of the scene is captured in quick succession, one using a phone-provided illumination and one without, keeping the exposure time and ISO fixed. The pixel values of the flash image are given by

$$\begin{array}{c} f^{F+A}=f^{F}+f^{A} \end{array}$$ (2)

where the values result from a sum of the two different sources present—flash, F, and ambient light A. The pixel values for the no-flash, or ambient, image are simply given by *f*^*A*^. It is important that the ambient lighting remains constant over the short time needed to capture the two images, else the contribution of *f*^*A*^ would change. Additionally, it is important that the response of the sensors across the three channels is linear with increasing intensity—doubling the intensity should double the pixel values. This linear response is expected over the mid-ranges of smartphone sensors. Where these conditions are met, it can therefore be seen that the subtraction yields pixel values influenced only by the flash illumination

$$\begin{array}{c} f^{F+A}-f^{A}=\left(f^{F}+f^{A}\right)-f^{A}=f^{F} \end{array}$$ (3)

The resulting data is under a standardised illumination, and hence data taken by the same device in different capture sessions can be directly compared. In the case of smartphone cameras, the ‘flash’ can be provided either by turning the screen backlight on and off in combination with the front facing camera or using the built-in LED flash with the rear facing camera.

#### Subtracted Signal to Noise Ratio (SSNR)

In order to avoid time-consuming recapturing of data, or loss of data, a metric to give an indication of whether the images captured are suitable is required at the time of capture. For the ambient subtraction method to yield good results, the flash must dominate over the ambient light. A simple intensity ratio of the flash to no-flash image seems to be a good option, however this does not take into account additional noise introduced if the overall signals are small. For a pixel value in the midrange of the sensor, as is typical when auto-exposure is used, shot noise dominates which can be described by a Poisson distribution [27]. In this case, the noise is simply given by the square root of the signal.

We suggest the Subtracted Signal to Noise Ratio (SSNR) as a suitable metric, given by the signal to noise ratio of the flash only pixel values obtained after subtraction

$$\begin{array}{c} \text{SSNR}=\frac{f^{F}}{noise(f^{F})}=\frac{f^{F+A}-f^{A}}{\sqrt{f^{F+A}+f^{A}}} \end{array}$$ (4)

where *f*^*F*+*A*^ and *f*^*A*^ are the pixel values recorded in the flash and no-flash images respectively, and the positive sign in the denominator is due to the summation of errors in quadrature [28]. To avoid introducing error from motion between images, this calculation is not performed pixelwise but instead the average signal for the region of interest is calculated for flash and no-flash images and a global SSNR calculated. Note that demosaiced images should be used to avoid biasing the results towards the green channel. The definition of the SSNR makes simplifying assumptions about sources of noise so that no other information is needed and the calculation can be based simply on the pixel values. In order to get a gauge of what the lower limit SSNR cutoff for ambient light-independent colorimetric measurements should be, an experiment was carried out. The results are presented later in this paper.

### Device independence

After performing ambient subtraction, results for a given phone are compatible over different lighting environments. However, to account for variations in the spectral sensitivity and spectral power distributions of different phone cameras and flashes, a conversion from phone native space to a device independent space is required. A reference space is defined as a color space which contains all possible colors and is device independent. The most common reference space is CIE XYZ space. This space was derived from the human visual system, with the aim of producing XYZ tristimulus values describing which combinations of light appear the same for a standard observer. Since its introduction in 1931, other spaces such as L*a*b* space have been designed for increased perceptual uniformity however these spaces are all based on transforms from XYZ space. The aim of this research is not to mechanize human color judgements but rather to obtain repeatable digital color descriptors that can then be linked to the application specific scale. Therefore, XYZ space has been used as a standard device independent color space. The conversion to this space accounts for variations in the phone spectral sensitivities, but it is also necessary to account for the different flash illuminations. To do this a set standard illuminant, here CIE D50, is chosen for the XYZ values. This means that XYZ values resulting from a conversion from two different phones should match.

The simplest way to achieve the conversion from phone native space to XYZ space is to utilise the commonly used open-source software dcraw [17]. The software uses information stored in the image metadata to do the conversion, but this information is optimised for images under a particular illumination and for a generic phone of that model. This means it does not take into account inevitable inter-device variations or the use of a different illumination [18]. An alternative approach is to use a colorcard such as the Macbeth ColorChecker Classic, which has 24 patches covering a wide range of colors and neutral shades. The XYZ values for each patch are provided, or can be measured with a spectrophotometer, and by capturing an image of the colorcard using each phone under the flash illumination a corresponding set of RGB values is produced. It is then possible to obtain a 3×3 device specific mapping, *M*, from native RGB to XYZ using a linear least squares approach

$$\begin{array}{c} M={\left(R^{T}R\right)}^{-1}R^{T}H \end{array}$$ (5)

where *R* and *H* are N×3 matrices of RGB and XYZ values respectively, and N is the number of patches [29]. A common alternative to the linear approach is to use a polynomial mapping, where *R* is expanded to include higher order power and cross terms in RGB [30]. To allow images to be taken at different illumination intensity levels without affecting the resulting colors, we require that the mapping used be exposure time independent. Since polynomial mapping terms are not raised to the same power, output values will be non-uniformly affected by a change in exposure time. A polynomial mapping would therefore not be appropriate here. A root polynomial approach is exposure time independent [31], but can lead to overfitting when only 24 patches are used. The simple linear mapping was therefore deemed most appropriate. Metamerism owing to the Luther condition not being met for the cameras means that the aim is always to find the closest approximation for the transformation to XYZ space from native space [32]. Note that the calibration stage to move from RGB to XYZ must be carried out on a per-device basis in order to achieve the higher accuracy, due to inter-device variation [18, 19, 33]. Whilst this may sound onerous, the use of this calibration step in combination with ambient subtraction means that it need only be carried out once—the mapping is optimised for the specific illumination provided by the phone which is always the resulting illumination after ambient subtraction. Data collection then becomes very simple, not requiring a colorcard to be included in each image.

#### Custom mapping

The use of a standard colorcard means that a variety of colors can be mapped with reasonable precision and accuracy, however in some cases the card may not yield precise enough results or may not cover the range of colors required. If the color in question is out of the gamut of the colorcard, the mapped value is likely to be highly inaccurate. Akkaynak et al suggested an approach for scene-specific color calibration which involves measuring the radiance of different parts of the scene and then calculating the corresponding XYZ values, as well as simulating the RGB values through additional calibration of the camera or obtaining them as above [19]. This approach is ideal for complex environments, such as underwater marine monitoring, however for more typical environments the additional complexity and specialised equipment required outweighs the benefit. The idea of developing a mapping which is targeted to a smaller range of colors, and which therefore maps them more precisely, remains desirable. In many colorimetric applications, the task is to discriminate between different levels of a particular color—for example red for anaemia [9], or yellow for jaundice [6]. In these cases the expected range of colors is known. It is therefore viable to create a physical custom colorcard with patches spanning the required colors. The use of a physical card means that the user does not need to have access to any equipment other than the colorcard and the phone they intend to use. Previous work on developing custom colorcards of this kind exists in areas such as agricultural plant monitoring [34], chronic wound monitoring [35] and characterising artwork [36]. We have taken the example of jaundice and have created a custom yellows card with the aim of increasing the accuracy of mapped yellow values. Experimental results demonstrating the concept are presented later in the paper.

### Chromaticity

Even after removing the effects of ambient light and moving to a device-independent space, the data from two phones will still not necessarily match. The image capture stage is flexible and hence photos may be captured at different distances and exposure times, meaning that the color channels will be subject to an unknown scaling factor. In addition, the region of interest within the image is likely to be affected by geometric shading, meaning that pixels which should have the same value will again be scaled differently. The approach to deal with these effects is to use chromaticity values rather than the raw channel values. Chromaticity is defined as a channel value divided by the sum of all three channels,

$$\begin{array}{c} x=\frac{X}{X+Y+Z},y=\frac{Y}{X+Y+Z},z=\frac{Z}{X+Y+Z} \end{array}$$ (6)

where lower-case letters are used to denote a chromaticity value, and any scaling factor across the channels will cancel out [22]. Chromaticity values sum to 1, meaning that our data has been reduced from three dimensions to two. After ambient subtraction, we apply the device-specific calibration and finally calculate device-independent x and y chromaticity values.

## Methods

In this section, the methodology for calibration and data collection are described, followed by a description of the experimental testing carried out to validate the overall method. Unless otherwise stated, all processing was carried out using MATLAB (MathWorks r2018a).

### Processing pipeline

#### One-time calibration

The first step when introducing a new phone for data collection is to carry out the one-time calibration required to make data from different phones compatible. Two flash/ no-flash pairs of images should be captured, one of the colorcard and one of a neutral grey card. The phone should be positioned at approximately 45° to the cards to minimise any specular reflection, and ambient light should be minimised. The grey card images are required to correct for the intensity non-uniformity of the smartphone flash. For this correction to work well, it is crucial that the phone remain as static as possible between the two sets of images. A low-cost phone tripod is ideal but where necessary, handheld measurements would be sufficient if done with great care. It was found that standard white printer paper is fairly non-uniform in reflectance and prone to specular reflection, hence the use of a grey card for the Intensity Non-Uniformity Correction (INUC).

**Fig 1:**
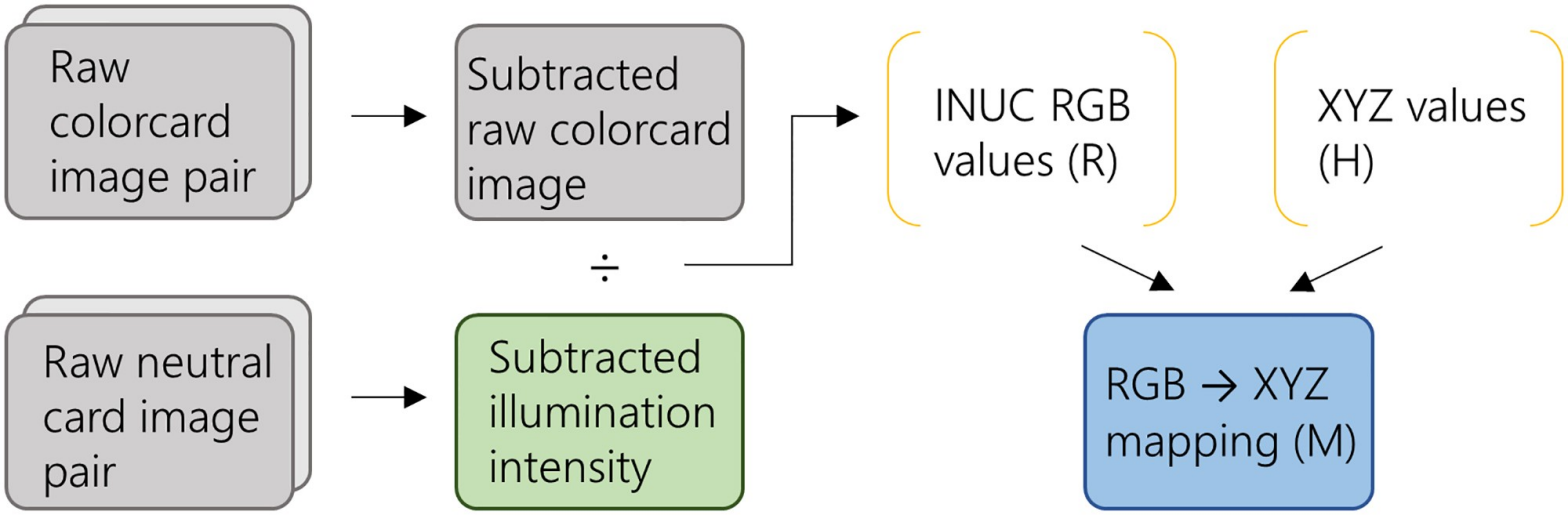
Calibration pipeline. The processing required to generate a device-specific RGB to XYZ mapping from flash no-flash image pairs captured of a colorcard and grey card with no ambient light. The subtracted RGB values for each colorcard patch are divided by the corresponding grey card intensity values obtained from the green channel to perform an Intensity Non-Uniformity Correction (INUC). The linear least squares mapping (M) is then constructed from known XYZ values (H) and the extracted RGB values (R). Note that due to the use of ambient subtraction this calibration needs only to be carried out once per device.

Once the images have been captured, the mapping from phone native space RGB values to device-independent XYZ values is carried out as depicted in Fig 1. The raw images are first linearised and subtracted. The grey card image is then demosaiced using dcraw (Dave Coffin version 9.27, 2016) and the green channel values selected as indicative of the overall intensity of the illumination. The extracted RGB values for each colorcard patch are then divided by the corresponding grey card intensity, and combined with the known XYZ values (provided by the card manufacturer or previously measured using a spectrophotometer) to give a mapping from RGB to XYZ, i.e. *H* = *RM*.

#### Data collection

**Fig 2:**
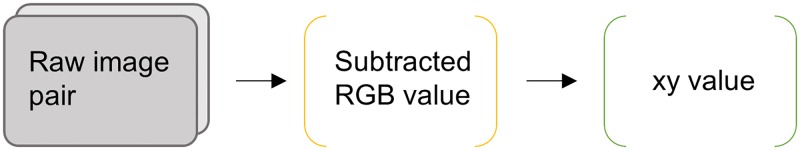
Data analysis pipeline. An image pair of the colorimetric subject is captured. The subtracted RGB value for the particular region of interest is then calculated by subtracting the median RGB values for each image. The previously calculated RGB to XYZ mapping (*M*) is then applied and xy chromaticity values are calculated, yielding device and ambient light independent color values.

Once the one-time calibration has been carried out, the data collection and following analysis process is extremely simple. Fig 2 shows the steps involved. First, an image pair containing the region of interest is captured, for example a patient’s eye or a test strip, ensuring that a good SSNR is obtained. Median RGB values for the particular region of interest are calculated for the flash and no-flash image, and the results are subtracted. This process accounts for any motion between images. The previously determined device-specific mapping (*M*) is applied to convert the subtracted RGB values into XYZ values. Finally the device and ambient light-independent xy chromaticity values are calculated. Datasets containing results from more than one phone are then compatible.

### Testing

In order to test the proposed methodology, two different models of phone were considered—the Samsung S8 and the LG Nexus 5X, referred to throughout the paper as simply S8 and Nexus. For image capture, the S8 rear camera was used with illumination provided by the LED flash, whereas the front-facing camera of the Nexus was used with a white screen as the illumination. Since it may be more useful to use either the front or rear-facing camera depending on the application, an example of both was considered. To investigate the variability of these phones within a specific model, two devices of each model were used. The linearity of the response of each phone to incident light was verified before use.

#### Ambient subtraction and SSNR threshold

The ambient subtraction method was tested for a wide range of colors by imaging 172 patches of the Macbeth ColorChecker DC card (excluding the repeating neutrals from the boundary of the chart and the reflective patches). Images of the DC card were captured with no ambient light, to provide a ground truth, and under daylight and fluorescent lighting. To evaluate this stage alone, the impact of ambient subtraction was considered in the phone native space by investigating the shift in rg values. Red and green chromaticity values, r and g respectively, were used to remove the effects of intensity and geometric shading.

The following experiment was carried out to provide guidance on the level of SSNR needed to produce useful data. The phone was held static at a 45° angle to a Macbeth ColorChecker Classic card, which has 24 patches, and flash/ no-flash image pairs were captured. A TaoTronics TT-DL09 LED desk lamp was used to provide a controlled ambient light with a correlated color temperature of 3850K, and image pairs were captured as the intensity was gradually increased. For each image pair the rg chromaticity values for each patch after subtraction were calculated and the distance to the corresponding ground truth rg values (*GT*), from a no ambient light image set, was calculated. The distance was defined as the Euclidean distance

$$\begin{array}{c} \text{rg}\;\text{distance}=\sqrt{{\left(r_{test}-r_{GT}\right)}^{2}+{\left(g_{test}-g_{GT}\right)}^{2}} \end{array}$$ (7)

where *r* and *g* are red and green chromaticities for the patches, and the *test* and *GT* subscripts refer to data under ambient and no ambient light respectively. This rg distance was then plotted as a function of the SSNR for each patch calculated according to Eq 4. The smallest rg distance achievable in practice was estimated by taking a series of no ambient light images of the Classic card over different capture sessions, and the average rg distance between the same patches imaged multiple times was calculated. This rg distance for each phone was then used to determine the suggested SSNR cutoff for practical use.

#### Device independence

The Macbeth ColorChecker Classic card (24 patches) was used for the one-time calibration for each phone, and then the testing was carried out using 148 patches from the Macbeth ColorChecker DC card (additionally excluding those out of the gamut of the Classic card). The ground truth xy chromaticity values for each card were measured using the X-Rite ColorMunki spectrophotometer. A procedure to calculate the Classification Accuracy for Multiple Phones (CAMP_*n*_, where *n* is the number of phones considered) was defined in order to compare our proposed overall pipeline to dcraw. Both mapping approaches were applied to the data and different subsets of the DC card patches were selected with varying allowed minimum separations in xy chromaticity space. The mapped values for each phone and patch were classified to the ground truth values using a nearest neighbour classification—the classification was deemed successful only if all four phones correctly classified a patch. In other words, to calculate the CAMP_4_ data presented later in the paper, these steps were followed:

- For a given minimum allowed xy separation
  - Select a subset of DC card points which are all at least this far apart
  - For each phone (1—*n*)
    - Do a nearest neighbour classification for all subset points of mapped to ground truth data
  - CAMP_*n*_ = percentage of points for which all phones gave the correct classification
  - Repeat for a minimum of 1000 unique point set permutations and find the average CAMP_*n*_

This classification process was carried out for image sets captured with no ambient light as well as for the image sets captured under daylight and fluorescent lighting, and the results compared. Finally, the concept of a custom colorcard was investigated for the example of yellows. Two custom cards were created, one for training and one for testing. The training card contained 30 patches covering yellows and neutrals and the testing card contained 24 patches of varying hue and saturation. The impact of using this custom card for mapping on the CAMP_4_ was compared to the Classic colorcard mapping approach for three different lighting conditions.

## Results and discussion

### Ambient subtraction

**Fig 3:**
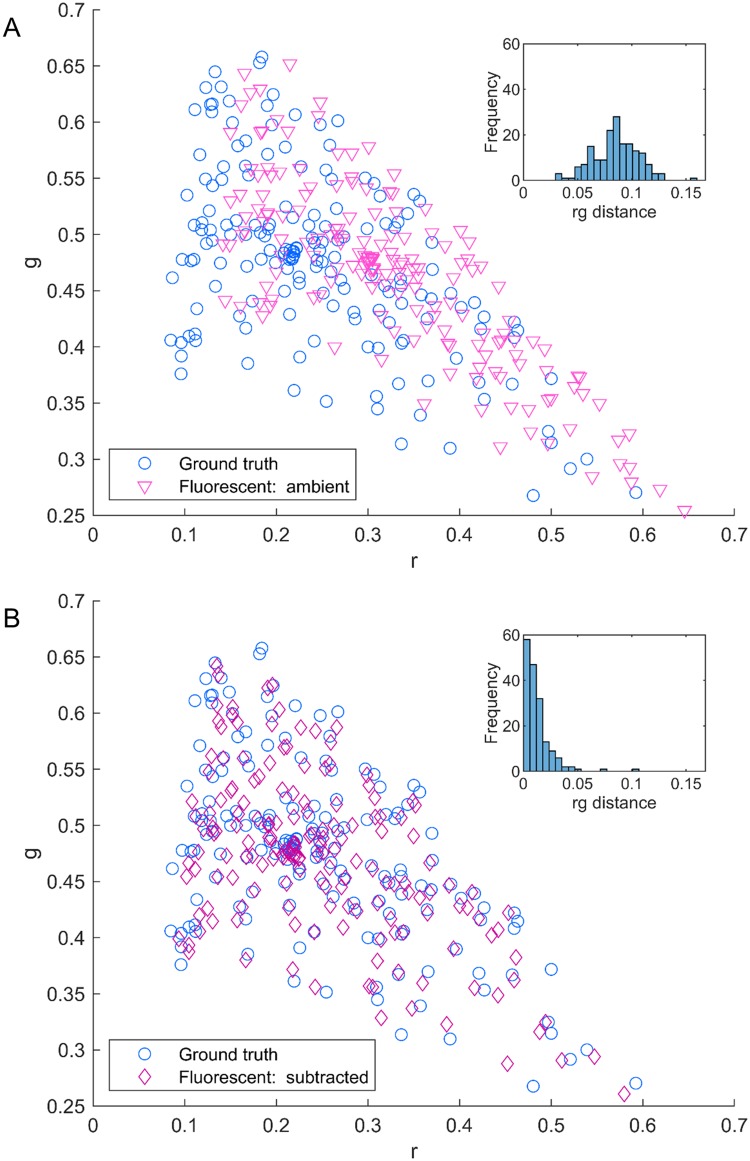
Impact of ambient subtraction. DC card rg chromaticity patch values for ambient fluorescent lighting are shown for an example Nexus phone before (A) and after (B) subtraction, as pale pink triangles and dark pink diamonds respectively. The ground truth rg values are denoted by blue circles, and the histogram of rg distances from the ground truth is shown as an inset in each subfigure. Note that after subtraction the match is greatly improved and the average rg distance decreases dramatically as the ambient subtraction minimises the effect of ambient light.

To test the ambient subtraction method, the DC card was imaged under daylight and fluorescent lighting. It was also imaged under no ambient lighting to provide a ground truth. Fig 3 shows the results before and after subtraction for an example Nexus phone under fluorescent lighting—results for all phones were similar in form, and fluorescent lighting results have been presented here as an example. From Fig 3 it is clear to see that the patch values after subtraction move towards the ground truth values, as the subtraction removes the impact of the ambient light on the pixel values. The average rg distance between corresponding pairs of ground truth and ambient light influenced values decreases significantly after subtraction, as demonstrated by the histograms inset in Fig 3.

**Fig 4:**
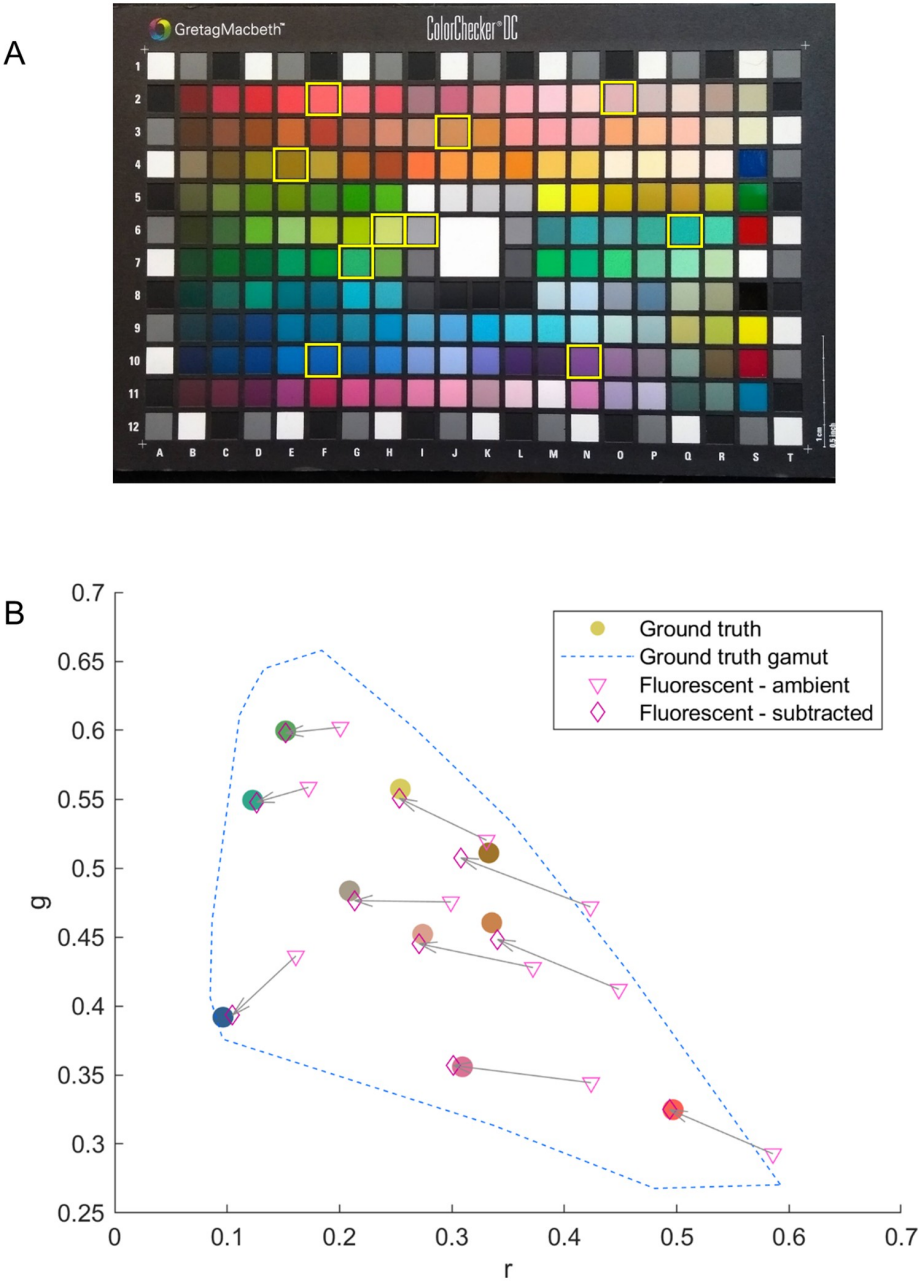
Visualisation of ambient subtraction. A subset of the example Nexus phone DC card patches shown in Fig 3 are shown to enable a clearer visualisation of the impact of ambient subtraction. An image of the DC card is shown in A with the selected patches outlined in yellow. The impact of ambient subtraction on these patches is shown in B. The outer limit of the ground truth rg values is shown with a blue dashed line and the subset of points have been selected to cover the gamut. The ground truth values are shown using large filled colored circles, where the circle color is given by the ground truth color of the patch. The values before and after subtraction are denoted by pale pink triangles and dark pink diamonds respectively, as before, and corresponding points are joined by an arrow. Note how in all cases the subtracted points move into close proximity of the ground truth value.

To aid a clearer visualisation of the impact of ambient subtraction, a subset of 10 points from the DC card is shown in Fig 4. The outer bounds of the total group of ground truth patch values from Fig 3 is shown with a blue dashed line, for reference. The subset of patches was chosen to be representative of the whole spread of points, with each ground truth value shown as a filled colored circle corresponding to the actual color of the patch. The values before and after subtraction are shown connected by an arrow. With the smaller set of points it is now even easier to see how much the ambient subtraction helps to standardise the data. The data presented here is again for fluorescent lighting, but similar results were found for daylight.

### SSNR

**Fig 5:**
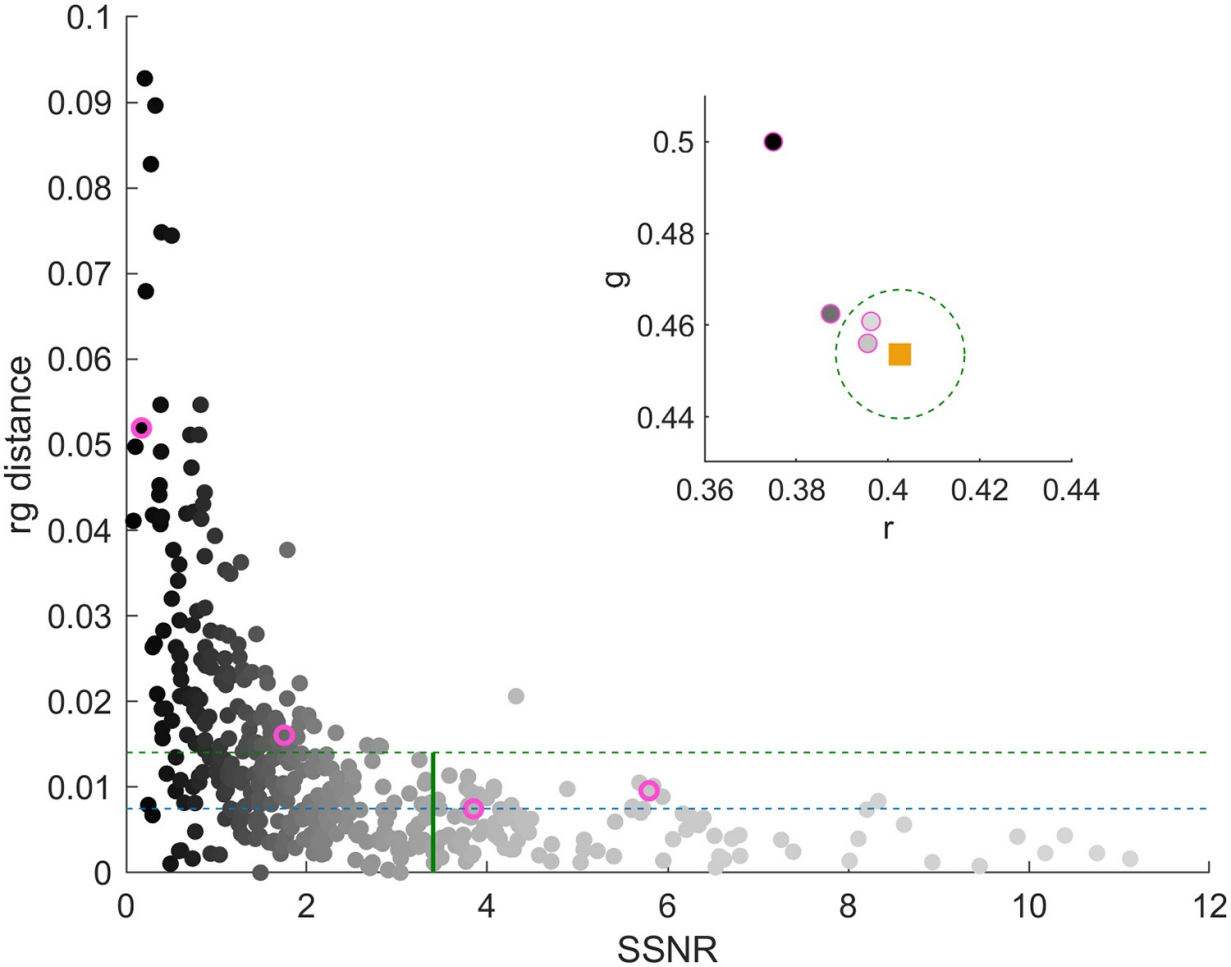
Determination of SSNR threshold. The Classic card was imaged under varying intensity ambient light. The rg distance of each patch from the ground truth, found using images under no ambient light, is plotted as a function of the SSNR value for the patch for an example S8 phone. The color of the points varies from black to pale grey according to increasing SSNR. The intrinsic threshold rg distance and the threshold plus one standard deviation are shown with dashed lines in blue and green respectively. The required SSNR for useable data is defined as the point at which all points are below the upper line, and therefore not limited in accuracy by SSNR. The overall experimentally determined SSNR threshold is 3.4, shown as a solid vertical green line. Additionally, an inset shows a subset of data points for an example patch. The square pale orange point represents the ground truth rg value for the selected patch, where the color of the square is given by the ground truth color of the patch. The green dashed line shows the threshold rg distance determined for adequate SSNR. Finally, the grayscale circles outlined in pink (as also outlined in pink in the main figure) show the initial large impact of increasing SSNR on the rg distance until the threshold where the results are comparable even for increased SSNR.

It is important to be able to know at the time of image capture whether an image set will be useable, to avoid loss of data. The SSNR gives information about the signal to noise ratio of the post-subtracted signal. However, there is not an intuitive value above which the images will be reliably useful. For this reason an experiment imaging the Classic colorcard under varying controlled levels of ambient light was carried out as described in the Methods section. Fig 5 shows the rg distance for each subtracted patch value from its corresponding ground truth value as a function of the SSNR for that patch for an example S8 phone. At a certain point, one would expect that increasing the SSNR would no longer improve (reduce) the rg distance significantly. This is evident in Fig 5, but it is hard to see where the rg distance stops improving. To provide a target rg distance threshold, the approach described in the Methods section was followed and the experimentally determined intrinsic rg distance error is shown in Fig 5 along with the value plus one standard deviation. For a practical limit, we deem that once all points are within this higher threshold they will not be limited by the SSNR. The inset of Fig 5 shows the ground truth rg value for an example patch along with some subtracted results with varying SSNR. The practical rg distance threshold is also marked in the inset, and it can be seen that above a certain SSNR the results remain within the threshold. For each phone in the study, the threshold SSNR was calculated using the baselines specific to that phone. The SSNR values yielded were similar for each phone and an average over the four phones results in a threshold SSNR of 3.4, marked in Fig 5. When capturing data using ambient subtraction, images should be retaken until the region of interest has an SSNR above this threshold.

### Device independence

For colorimetric applications, it is important to establish how well phones agree with each other as well as how precise the results are. For certain applications, it will only be necessary to discriminate between a few very different colors, but some will have many more. In order to test our approach, the CAMP_4_ was calculated for subsets of DC card patches with a range of minimum xy separations as described in the Methods section.

**Fig 6:**
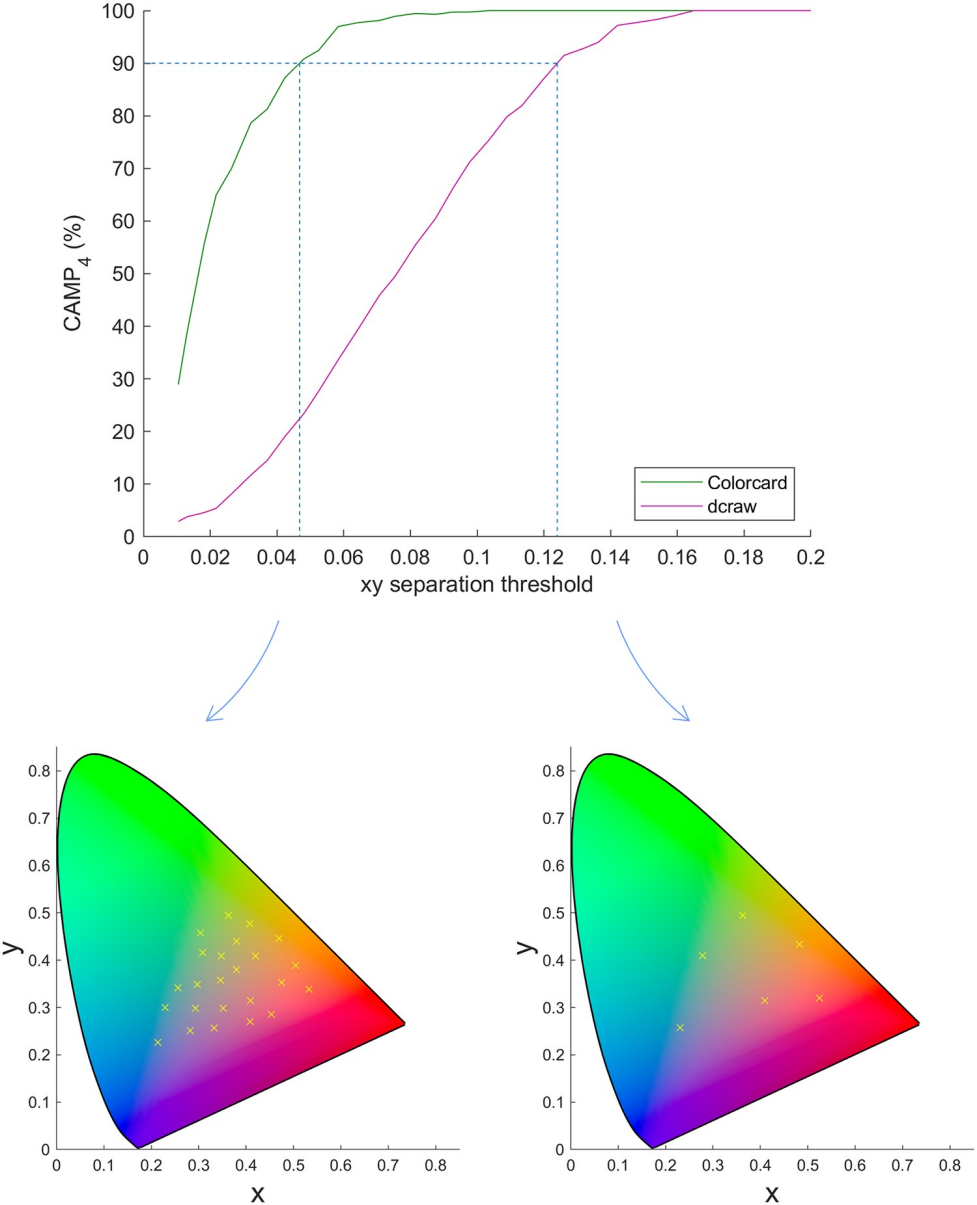
Classification accuracy. CAMP_4_ (Classification Accuracy for Multiple Phones, 4 phones) for different subsets of patches from images of the DC card under fluorescent illumination for our proposed method and dcraw are shown as a function of the average minimum xy separation. Note the large increase in classification accuracy for our approach at lower xy separations compared to dcraw, the simplest alternative. The blue dashed lines indicate the xy distance at which a 90% CAMP_4_ is achieved for each method, with corresponding example DC patch subsets shown below to enable a visual understanding of different colors that can be discriminated between with the two approaches. xy chromaticity diagrams generated using [37].

Fig 6 shows the CAMP_4_ of both approaches over a range of average minimum xy separations for images captured under fluorescent lighting. It is clear from Fig 6 that our approach provides higher accuracy for smaller point separations—to achieve a 90% classification accuracy the xy separation required for the dcraw approach is over 0.12, whereas this level is reached for our approach at xy separations of under 0.05. Results were very similar for all three lighting environments, with a spread in the xy separation required for 90% accuracy for the different approaches of less than 0.01, highlighting further the power of ambient subtraction. Example subsets of the DC patches are shown below the main figure for separations yielding a 90% CAMP_4_ for the two methods. Note how much more similar colors are classified correctly for our approach. For applications where it is only necessary to discriminate between a few very different colors, the required accuracy may be provided by using dcraw or an alternative method using the metadata information. However, when more similar colors are included then our approach provides a significant increase in classification accuracy. Note that for this increase in accuracy to be maintained, it is important to use a device-specific calibration. If instead a model level calibration level is used, for example one calibration for all S8 phones, the accuracy will be reduced [18]. However, since this device-specific calibration need only be carried out once per device it is not too onerous.

### Custom mapping

**Fig 7:**
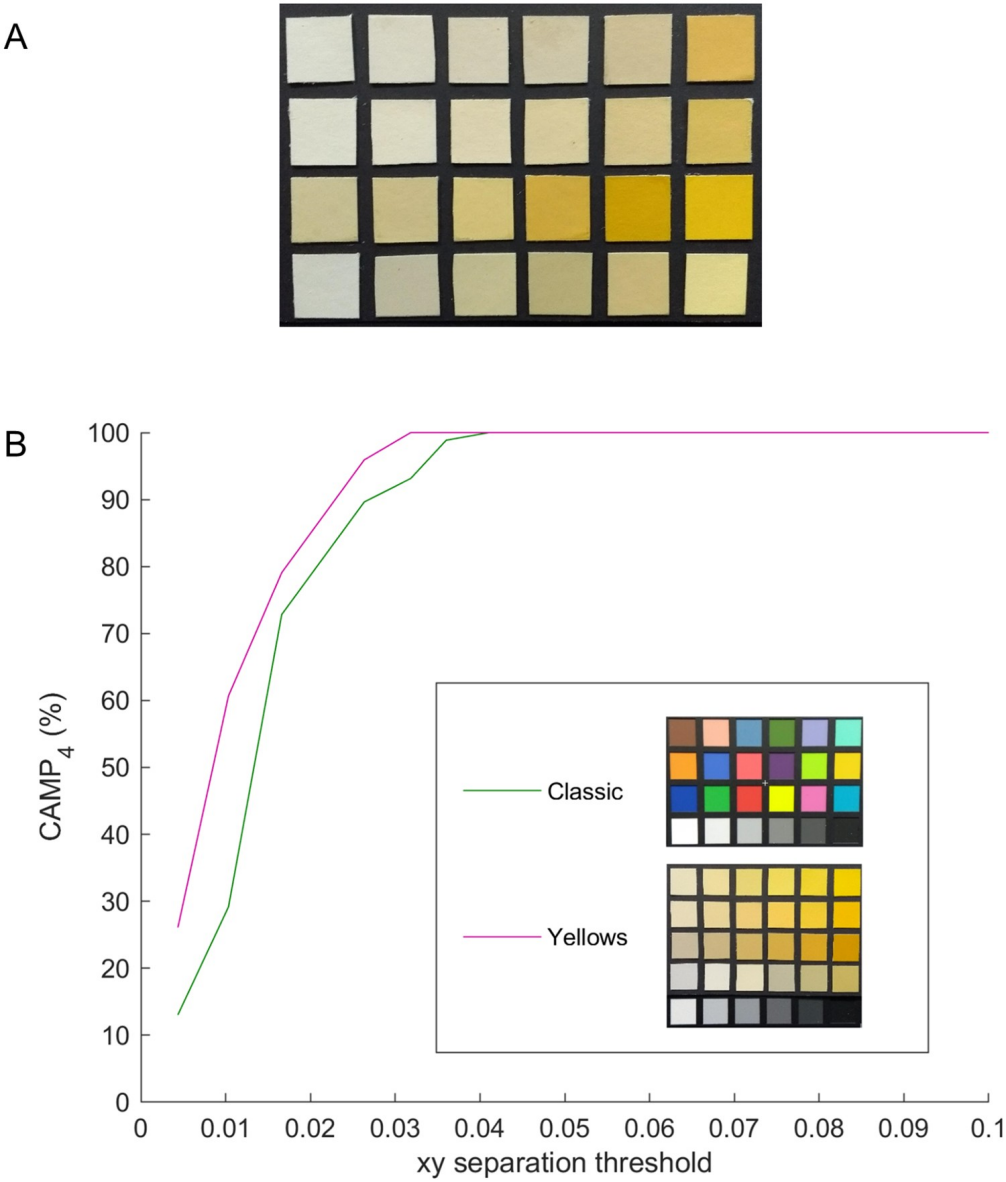
Custom mapping classification accuracy. An image of the yellows card used for testing is shown in A with the CAMP_4_ (Classification Accuracy for Multiple Phones, 4 phones) shown in B. Results are presented for mappings developed using the Classic card and training yellows card (with images of the cards in the legend) as a function of the average minimum xy separation for different point subsets of the test yellows card with no ambient light. Note the increase in classification accuracy for small xy separations when the yellows card approach was used, crucial when trying to discriminate between very similar colors.

As previously discussed, the two scenarios where it may be helpful to use a custom colorcard are when the colors of interest are out of the range of the Classic card, or when the level of precision and accuracy required in a small color region is very high. Here we consider the second case, and take the specific example of yellow—high accuracy is required specifically for levels of yellow when quantifying jaundice. The impact of calibrating using the Classic card versus a custom yellows card was tested using a second yellows card, with patches from a different manufacturer and with different reflectance profiles. The same process for finding the CAMP_4_ of the two methods was used as for the more general classification testing. Fig 7 shows images of the cards used and the CAMP_4_ as a function of the mean minimum xy separation for the subsets with no ambient light. The yellows card approach provides a higher classification accuracy than the Classic card for all xy separations, with a particularly large difference for small xy separations. The precision of the mapping is also important, particularly when focussing on a small region of color space. Table 1 gives the overall mean, median and 95th percentile xy error distance over the four phones for the two methods. Using the yellows card for the one-time calibration rather than the Classic card leads to an increase in accuracy of around 40% based on the median. The aim of this example case was to demonstrate that an increase in both pure mapping accuracy and CAMP_4_ can be achieved when using a custom colorcard which focusses on a smaller range of colors than the Classic card. For a given application, a custom card could be designed and used for the calibration stage, leading to more precise color information whilst maintaining a simple calibration process.

**Table 1:** Accuracy of Classic and custom mappings on test yellows card.

Mapping approach	xy error distance		
	Mean	Median	95th percentile
*Classic*	0.006	0.005	0.013
*Yellows*	0.003	0.003	0.010

Average mean, median and 95th percentile xy error distances of the mapped values to the ground truth test yellows card values for data under no ambient light. Data is presented for the calibration carried out using the Classic colorcard and the custom yellows card.

## Conclusion

Through the use of our novel processing pipeline for smartphone colorimetric measurements, the effects of ambient light and inter-device variation can be accounted for and reliable chromaticity values obtained. Capturing pairs of flash/ no-flash images and using an ambient subtraction technique minimizes the effect of ambient light. Then the application of a device-specific mapping, developed from images of a standard colorcard, allows the conversion of values to a device-independent space such as xy space. The extracted color values are then independent of the device and lighting. This means that the link from chromaticity values to the physical scale relevant to the particular application only needs to be developed once and can be applied to data collected using new phones. Additionally, we propose the use of a physical custom colorcard for applications where more precise values are required. When the mapping is focussed on a smaller region of colorspace the results can be more accurate, and the use of a physical card means that the simple one-time calibration can still be carried out by imaging the card.

One potential drawback of our proposed method is that it yields just chromaticity color values, reduced by one dimension. For many applications this is not a problem, however in some cases crucial information is lost when the dimensionality is reduced. It would be possible to expand to the full colors by including a white standard in each image, however we have avoided this due to our aim of keeping the image capture simple. The latest generations of smartphones are starting to include time of flight cameras which could be integrated into the method to provide distance information and so normalise the data and enable the full color values to be extracted.

The one-time calibration method laid out here involves imaging a grey card to correct for variations in the illumination intensity across the colorcard. Moving forwards, it may be possible to further simplify this process by imaging only the colorcard and applying the alternating least squares color correction algorithm proposed by Finlayson et al [38]. This algorithm corrects for the spatial variation and generates the mapping by alternating between the two steps. Initial tests using the approach are promising, and could make the calibration process even more simple.

Our color science-based approach to smartphone colorimetry enables image capture using multiple phones in different environments. The processing power required is low and so eventually the entire system could be integrated within an app which would not require cloud access, making the approach fit for use in low resource or remote environments. Additionally, the one-time calibration and straightforward image capture process make our method a simple, streamlined approach for smartphone colorimetric measurements.
